# Urea transporters and sweat response to uremia

**DOI:** 10.14814/phy2.12825

**Published:** 2016-06-07

**Authors:** Raymond W. Keller, James L. Bailey, Yanhua Wang, Janet D. Klein, Jeff M. Sands

**Affiliations:** ^1^ Renal DivisionDepartment of MedicineEmory University School of MedicineAtlantaGeorgia; ^2^ University of New EnglandCollege of Osteopathic MedicineBiddefordMaine; ^3^ Department of PhysiologyEmory University School of MedicineAtlantaGeorgia

**Keywords:** Chronic renal failure, dialysis, end stage renal disease, sweat, urea

## Abstract

In humans, urea is excreted in sweat, largely through the eccrine sweat gland. The urea concentration in human sweat is elevated when compared to blood urea nitrogen. The sweat urea nitrogen (UN) of patients with end‐stage kidney disease (ESRD) is increased when compared with healthy humans. The ability to produce sweat is maintained in the overwhelming majority of ESRD patients. A comprehensive literature review found no reports of sweat UN neither in healthy rodents nor in rodent models of chronic kidney disease (CKD). Therefore, this study measured sweat UN concentrations in healthy and uremic rats. Uninephrectomy followed by renal artery ligation was used to remove 5/6 of renal function. Rats were then fed a high‐protein diet to induce uremia. Pilocarpine was used to induce sweating. Sweat droplets were collected under oil. Sweat UN was measured with a urease assay. Serum UN was measured using a fluorescent ortho‐pthalaldehyde reaction. Immunohistochemistry (IHC) was accomplished with a horseradish peroxidase and diaminobenzidine technique. Sweat UN in uremic rats was elevated greater than two times compared to healthy pair‐fed controls (220 ± 17 and 91 ± 15 mmol/L, respectively). Post hoc analysis showed a significant difference between male and female uremic sweat UN (279 ± 38 and 177 ± 11 mmol/L, respectively.) IHC shows, for the first time, the presence of the urea transporters UT‐B and UT‐A2 in both healthy and uremic rat cutaneous structures. Future studies will use this model to elucidate how rat sweat UN and other solute excretion is altered by commonly prescribed diuretics.

## Introduction

Human sweat is known to contain solutes that accumulate in patients with renal failure. In particular, urea concentrations in human sweat are persistently elevated in patients with increased blood urea nitrogen levels (Huang et al. 2002). Perhaps, the most stunning manifestation of this phenomenon is the uremic frost (Pol‐Rodriguez et al. 2008; Saardi and Schwartz 2016), in which urea crystals form on the skin of patients in a distribution consistent with the sweat glands. This is usually visible on the glabella or other body surfaces that are not washed frequently nor subject to forces which rub the urea off. When uremic frost appears, it is an indication of uremia and is a harbinger of kidney failure. Although impressive, it is only rarely seen as kidney failure is usually diagnosed early in the disease progression and dialysis lowers blood urea levels sufficiently to prevent uremic frost.

Urea transport across lipid bilayers has classically been attributed to simple diffusion. We now know that urea transport across cell membranes is facilitated by urea transporters (UT) (Sands 2003). Since their discovery, UT's have been found in a variety of human tissues, including the eccrine sweat glands (Jing et al. 2013). Our lab has previously demonstrated the presence of UT‐B in human eccrine clear cells (R. W. Keller, unpubl. obs.). It is likely that these transporters play a role in urea secretion in sweat.

Humans sweat to thermoregulate, an ability that is rare in the animal kingdom. Rats do not sweat in response to elevation of core body temperature to at least 41°C (R. W. Keller, unpubl. obs.), but do sweat in response to pilocarpine and other cholinergic agonists. Humans are also unique in their almost total body distribution of sweat glands. Rodents possess sweat glands only on their footpads. These glands are classically ascribed the role of increasing traction, a function homologous to the eccrine glands of human volar surfaces.

In this study, we sought to determine if the urea levels in rat sweat rise with an increase in blood urea nitrogen levels in the setting of renal insufficiency. Additionally, we wondered if urea transporters were present in the volar skin of the rat footpad. We hypothesized that sweat from uremic rats would contain a higher concentration than their pair‐fed controls. Lastly, we questioned whether UT's would be present in the footpad eccrine glands.

## Methods

### Animals

Sprague–Dawley rats weighing between 200 and 250 g (8 male and 13 female) were used in this study. Half of each gender served as pair‐fed nonnephrectomized controls. All work was approved by the Emory Institutional Animal Use and Care Committee.

### 5/6 nephrectomy

To prepare the chronic renal failure model, renal artery ligation of the right kidney followed by unilateral nephrectomy was performed. The rat was allowed to recover for 1 week, then the upper and lower segmental renal artery ligation was utilized to infarct 2/3 of the remaining kidney (Bailey et al. 1995). During the first 7 days of recovery, animals were given free access to a 14% low‐protein diet (Teklad Global 2014, 14% Protein Rodent Maintenance Diet) and provided with ¼ normal saline (0.23 mol/L NaCl) to drink. Following the recovery period, animals were maintained on normal (23%) protein diet (Lab Diet 5001, Teklad) until induction of uremia.

### Inducing uremia

To induce uremia, rats were fed a 40% protein diet (Lab Diet 5001 with added casein) ad libitum while continuing 0.23% saline drinking water. Food intake was monitored daily and the control rats were pair‐fed the same amount of food consumed by the uremic rats to maintain comparable levels of protein intake. Animals were observed daily for signs of uremia, including weight loss, spiky fur, anorexia, or lethargy, and uremia was confirmed by measuring serum urea levels postmortem in a 1/3 of the rats.

### Sweat induction and collection

Rats were anesthetized with ketamine/xylazine; then, the rat footpads were cleaned with three washes of distilled water. Footpads were immersed in light mineral oil to prevent evaporative losses. Pilocarpine (10 mg/kg) in water was injected subcutaneously dorsal to the scapula to induce sweating. A 20–70 nL constriction pipette was used to collect sweat, with mineral oil maintaining a vapor barrier at all times. Sweat was then analyzed for urea content.

### Urea assay

The urea concentrations were measured enzymatically using a continuous flow ultramicrofluorometer (Sands and Knepper 1987). The two enzymatic reactions are: (1) conversion of urea to ammonia catalyzed by urease; and (2) reaction of the ammonia with a‐ketoglutarate and NADH to form NAD, catalyzed by glutamate dehydrogenase. The reagents were purchased as a kit (Kit 65‐A, Sigma, Saint Louis, MO). Urea presence in the sample is proportional to the disappearance of NADH, measured as the reduction from base‐line fluorescence.

### Immunohistochemistry

Immediately after death, footpads were placed in 10% neutral‐ buffered formalin for fixation. After 2 days of fixation, the footpad was degloved and the skin was placed in a 30% sucrose solution for cryoprotection. After 3 days in sucrose, the skin was frozen in liquid nitrogen. The tissue was sliced into 7 *μ*m sections and mounted on slides for staining. Slides were incubated at 4°C overnight with primary antibodies to UT‐A1 (1:2500), UT‐A3 (1:1500), or UT‐B (1:2500). Visualization was achieved using the standard ABC kit (Vectastain PK‐4000) according to the manufacturer's instructions.

### Statistical analysis

Sweat UN levels are reported as the mean ± standard error of the mean (SEM), with the number of samples reported for each group. Prism (GraphPad Software, Inc., La Jolla, CA) was used for two‐ and one‐way analysis of variance (ANOVA). Post hoc Tukey's range test was used to determine the difference between uremic male and female sweat urea nitrogen (UN) values. The criterion for statistical significance was *P *<* *0.05.

## Results

### Sweat urea nitrogen

**Figure 1:**
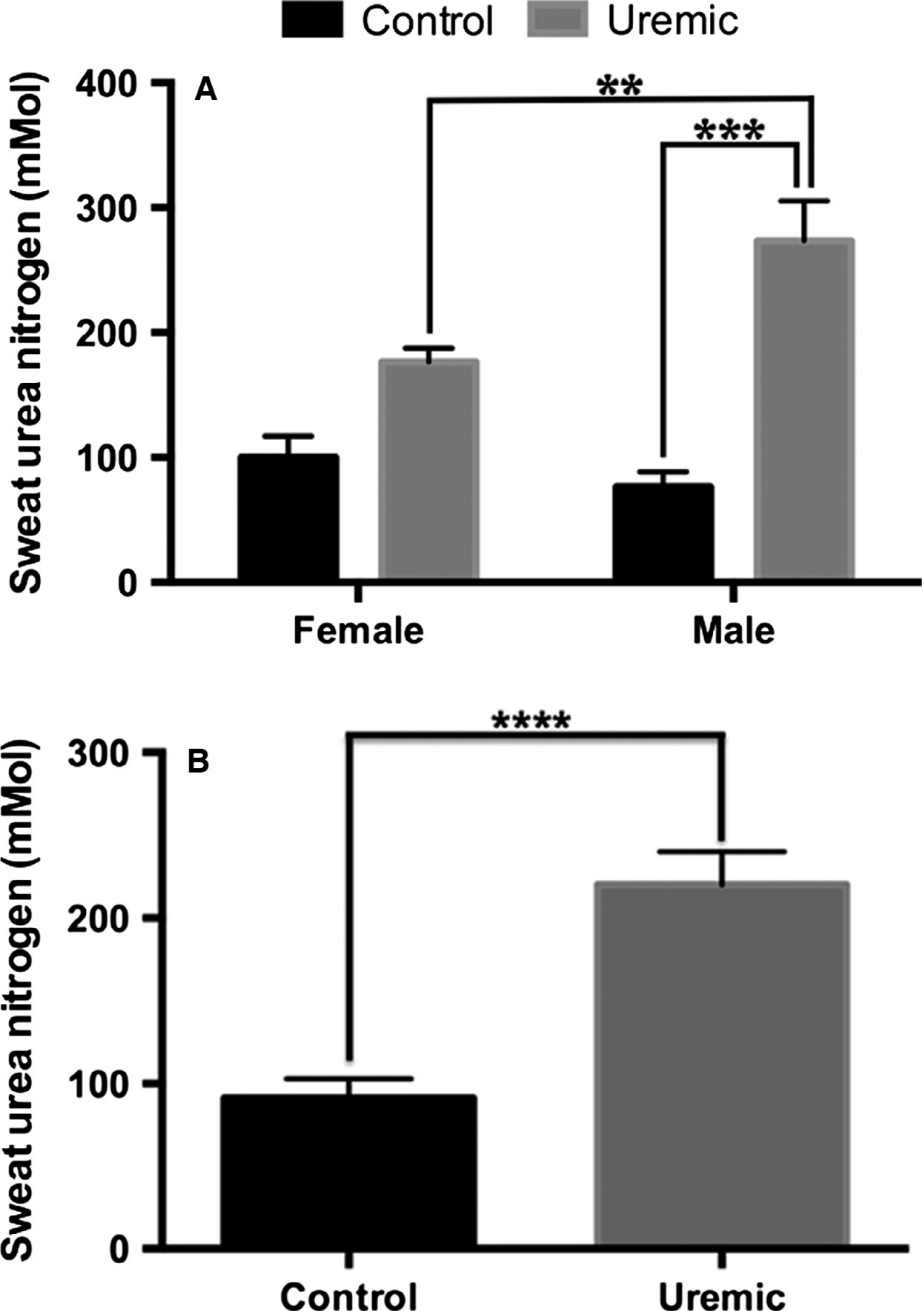
Sweat urea nitrogen levels from (A) Healthy control and 5/6 nephrectomized uremic rats separated by gender. Significance at *P* < 0.05 (**) and *P* < 0.01 (***). (B) Healthy control and 5/6 nephrectomized rats not separated by gender data with significance at *P* < 0.0001(****).

Four groups of rats were compared: (1) male controls (MC); (2) male uremics (MU); (3) female controls (FC); and (4) female uremics (FU). Average ± SEM sweat urea nitrogen in mmol/L for each group were: MC 77 ± 12, MU 287 ± 37, FC 100 ± 17, FU 175 ± 11 (*n* = 3,5,5,8, respectively). Figure 1A shows 2‐way ANOVA with Tukey's multiple comparison. There is a significant difference between male and female uremic rats compared to their gender‐matched controls, but not between female controls and female uremic rats. However, there was a trend toward significance when comparing the uremic females to the female controls. Figure 1B shows one‐way ANOVA of all uremics versus all controls, regardless of gender. This analysis shows a significant difference between all uremic rats and controls (220 ± 17 and 91 ± 15 mmol/L, *n* = 13 and 8, respectively). Protein intake did not correlate with sweat urea levels between any of the groups above (data not shown).

### Immunohistochemistry

**Figure 2:**
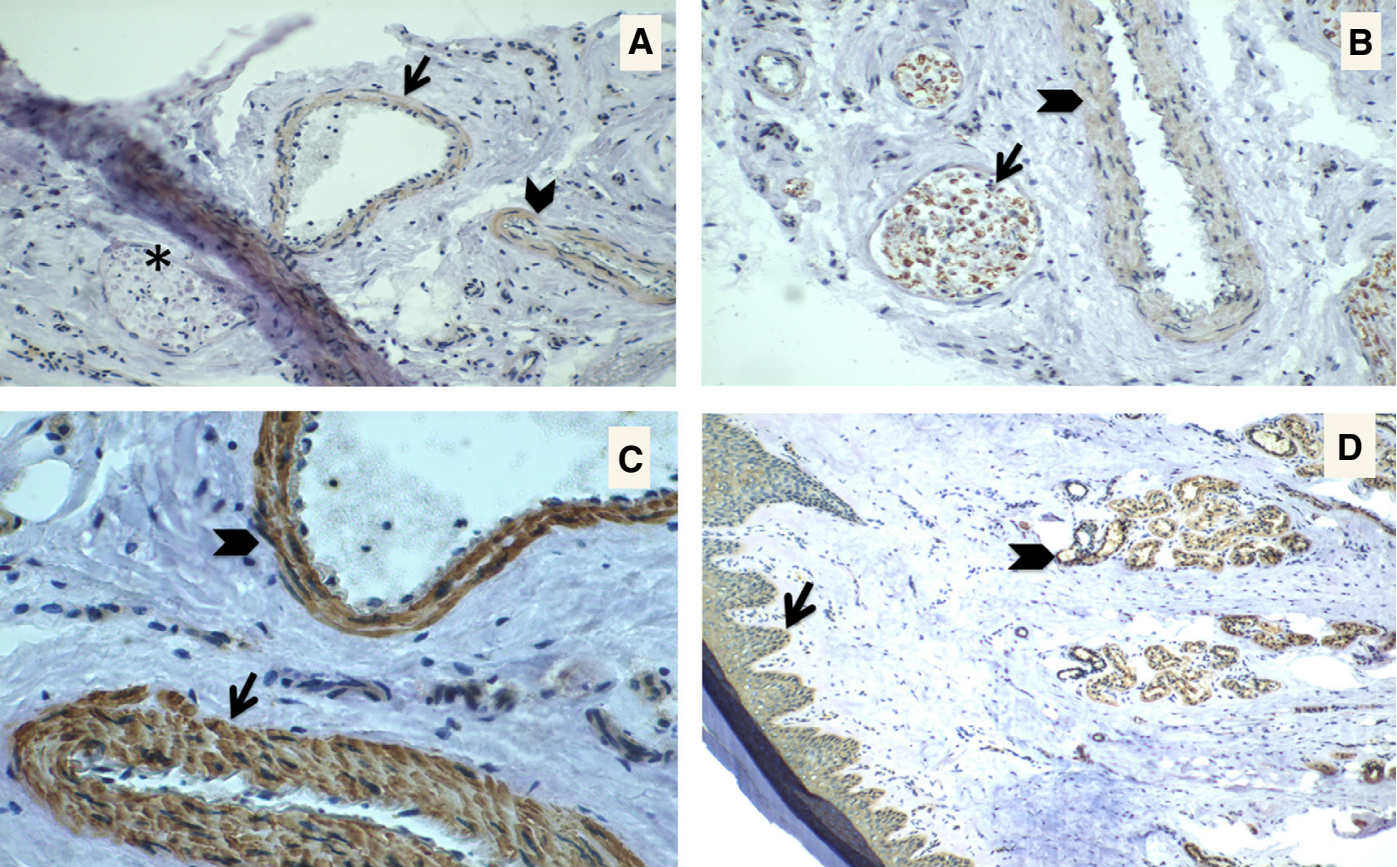
Immunohistological analysis of footpad skin from 5/6 nephrectomized rats for UT‐A N‐ and C‐terminus, and UT‐B. Panel A: UT‐A N‐terminus antibody staining in peripheral artery (arrow) and vein (arrowhead), asterisk is over peripheral nerve (not positive staining). 20× magnification, PAS. Panel B: C‐terminus staining in peripheral nerve cells (arrow) and peripheral artery (arrow head). Panel C: UT‐B staining in cutaneous artery (arrow) and cutaneous vein (arrowhead). 40×, PAS. Panel D: UT‐B staining in epidermis (arrow) and sweat gland secretory coil (arrowhead). 10×, PAS.

Figure 2 shows the results of IHC. UT‐B is expressed in the eccrine secretory coil and all layers of the epidermis. UT‐B staining was also positive in all layers of the large veins and arteries of the footpad. Staining of the footpad skin with antibody to UT‐A N‐terminus identified peripheral artery and vein. This antibody did not stain peripheral nerve. The antibody to UT‐A C‐terminus identified peripheral nerve cells and peripheral artery (Fig. 2B). The staining of both N‐terminus (Fig. 2A) and C‐terminus (Fig. 2B) suggests that UT‐A1 was present in the large cutaneous arteries and veins. The absence of positive staining of nerve with N‐terminus antibody in Figure 2A but positive staining with C‐terminus antibody (Fig. 2B) suggests that UT‐A2 staining was present in the cutaneous peripheral nerve bundles associated with large vessels.

## Discussion

This study suggests that the elevated level of urea in uremic rat sweat is similar in magnitude to the elevation observed in human sweat (Henderson et al. 1982). Thus, this model may serve as a way to study excretion of urea in the sweat of rats. Furthermore, other similarities between rat and human sweat may be found that allow study of excretion of other substances such as potassium, beta‐2 microglobulin, or indoxyl sulfate.

The sex difference in sweat UN was a surprising finding. In humans, the menstrual cycle is known to change the characteristics of thermoregulation and electrolyte balance. Estrogen lowers core body temperature at rest and during heat stress (Tankersley et al. 1992). Additionally, estrogen replacement therapy increases total body sodium. The estrous phase of our rats was unknown. Future work could be undertaken to elucidate the relationship between estrogen and urea excretion in sweat.

The obvious limitations of this study are threefold. First, the sweat produced in rodents is done with a cholinergic agonist and, at least in humans, there are differences between pharmacologic and thermal sweat compositions. Pharmacologic sweat is higher in potassium and usually higher in sodium than thermal sweat (Sato et al. 1970). We are unaware of studies investigating a similar phenomenon for urea secretion in humans or animals.

Second, rodents are likely unable to produce enough sweat volume to significantly alter their whole body urea, electrolyte, or water balance. This is due to the fact that rodents only possess 800–1600 eccrine glands per animal, compared to 1.6–5 million in humans (Wilke et al. 2007). Although one report of mice sweating in response to heat has been reported (Tian et al. 2007), we have not achieved this response in rats. Even if a rat could be made to sweat for 24 hours, an optimistic sweat volume would be 2 mL/day. This is compared to average daily urine outputs of 13 mL for healthy and 47 mL for uremic rats (Kumano et al. 1986).

Third, rodents lack the dark cells characteristic of human eccrine glands. The dark cells are classically associated with mucopolysaccharide secretion, while the clear cells are responsible for the serous portion of sweat. This study demonstrates that urea is excreted by clear cells, but does not rule out the possibility that dark cells may also contribute to urea excretion.

Because humans have the ability to produce a voluminous sweat, whole body balances of sodium and potassium, and likely urea, can be affected. Small studies have demonstrated that sweat therapy can lower plasma levels of urea and potassium in patients with renal insufficiency (reviewed in [Ting et al. 2014]). It is tempting to speculate that, with the proper medical oversight, sweat therapies could provide therapeutic benefits to patients with renal disease.

## Conflict of Interest

None declared.
